# Carboxymethyl Cellulose (CMC) Optical Fibers for Environment Sensing and Short-Range Optical Signal Transmission

**DOI:** 10.1021/acsami.1c22227

**Published:** 2022-01-08

**Authors:** Aayush Kumar Jaiswal, Ari Hokkanen, Markku Kapulainen, Alexey Khakalo, Nonappa, Olli Ikkala, Hannes Orelma

**Affiliations:** † Biomaterial Processing and Products, VTT Technical Research Centre of Finland Ltd., Tietotie 4E, 02044 Espoo, Finland; ‡ Microelectronics, VTT Technical Research Centre of Finland Ltd., Tietotie 3, 02044 Espoo, Finland; § Faculty of Engineering and Natural Sciences, Tampere University, P.O. Box 541, 33101 Tampere, Finland; ∥ Department of Applied Physics, Aalto University, P.O. Box 15100, 00076 Espoo, Finland

**Keywords:** cellulose, optical fibers, sensors, respiratory sensors, green photonics, biosensors

## Abstract

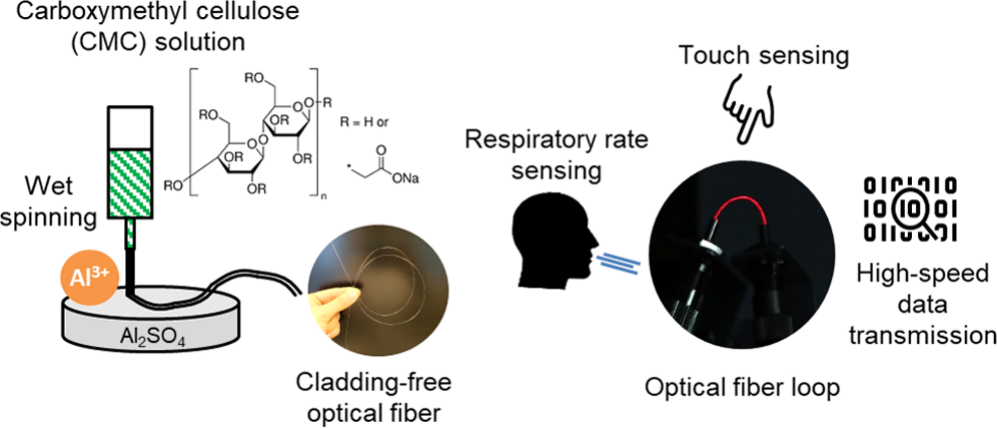

Optical fibers are a key component in modern photonics, where conventionally used polymer materials are derived from fossil-based resources, causing heavy greenhouse emissions and raising sustainability concerns. As a potential alternative, fibers derived from cellulose-based materials offer renewability, biocompatibility, and biodegradability. In the present work, we studied the potential of carboxymethyl cellulose (CMC) to prepare optical fibers with a core-only architecture. Wet-spun CMC hydrogel filaments were cross-linked using aluminum ions to fabricate optical fibers. The transmission spectra of fibers suggest that the light transmission window for cladding-free CMC fibers was in the range of 550–1350 nm, wherein the attenuation coefficient for CMC fibers was measured to be 1.6 dB·cm^–1^ at 637 nm. CMC optical fibers were successfully applied in touch sensing and respiratory rate monitoring. Finally, as a proof-of-concept, we demonstrate high-speed (150 Mbit/s) short-distance signal transmission using CMC fibers (at 1310 nm) in both air and water media. Our results establish the potential of carboxymethyl cellulose-based biocompatible optical fibers for highly demanding advanced sensor applications, such as in the biomedical domain.

## Introduction

Since their invention in the 1960s, optical fibers (OFs) have become a key component in telecommunication, data transmission, sensing, and illumination. Initial optical fibers were based on silica glass, termed glass optical fibers (GOFs), and their application as a light waveguide was realized owing to their extremely low attenuation (∼0.2 dB·km^–1^ in the 1500–1600 nm range).^1,2^ The total transmission range of a common silica-based GOF is from 300 to 2000 nm, and it can be increased from 2 to over 10 μm using special glass dopants like fluoride and chalcogenide.^3^

As an alternative to GOFs, polymer optical fibers (POFs) are predominantly manufactured from resins such as polystyrene (PS), polycarbonate (PC), and poly(methyl methacrylate) (PMMA). These organic materials exhibit a low-attenuation high-transmission window in the visible wavelength range (400–700 nm), are flexible and bendable, and allow the production of fiber with a larger diameter (∼1 mm). For instance, commercial POFs made of PS, PC, and PMMA exhibit attenuation values of approximately 330 dB·km^–1^ (570 nm), 600 dB·km^–1^ (670 nm), and 55 dB·km^–1^ (538 nm), respectively.^4^ Evidently, attenuation values for POFs are 2–3 orders of magnitude higher than commercial GOFs, and thus, their applications are limited to shorter distance end uses such as automobiles, medical devices, and decorative illumination.^5^ A major drawback of POF sensors is their low operating temperature range, which is caused by glass transition in polymer materials. It is typically below 80 °C for PMMA and below 125 °C with PC.^4^

Both GOFs and POFs are not only excellent candidates in data transmission applications, but their use in sensing applications is also widespread. The key benefits of using OF-based sensors include their immunity to electromagnetic interference, small size, high sensitivity, large bandwidth, and ability to provide distributed sensing.^6^ Major measurands for optical fiber sensors are strain, temperature, and pressure, where these sensors are typically based on fiber Bragg gratings (FBG),^7,8^ ring interferometry,^9,10^ and scattering or reflection changes.^11^ In addition to these conventional measurands, optical fiber sensors have also been shown to detect humidity,^12^ displacement,^13^ and chemical composition.^14^ In biomedical applications, for instance, agarose-infiltrated photonic crystal GOFs have been utilized for breath monitoring during magnetic resonance imaging (MRI) analysis.^15^ Such optical interferometric reflection type sensor is required because it is not possible to put electrical sensors inside MRI systems. Elsewhere, a fiber optic sensor was demonstrated for the treatment of Parkinson’s disease in mice.^16^

Recently, due to concerns relating to environmental sustainability, biopolymer-based OFs, such as cellulose and its derivatives, have gained attention. Cellulose, being a renewably sourced biopolymer, offers several advantages over conventional materials, such as biocompatibility, aqueous processing, and biodegradability at the end-of-life.^17,18^ Moreover, utilization of cellulosic materials also lowers the dependence on nonrenewable and heavy greenhouse emission-causing petroleum resources for plastic materials. Orelma et al. were the first to report the use of cellulose fiber as a waveguide where they prepared OFs using cellulose regenerated from 1-ethyl-3-methylimidazolium acetate [EMIM]OAc as the core and cellulose acetate as cladding.^19^ Using a similar architecture, Reimer et al. recently reported OFs made from cellulose regenerated from *N, N*-dimethylacetamide (DMAc) and lithium chloride (LiCl) with cellulose acetate derivatives as cladding materials.^20^ Hynninen et al. presented a regeneration-free approach to prepare OFs based on methylcellulose (MC), where OFs were prepared from MC hydrogels.^21^ Moreover, gold nanoclusters were incorporated into the MC fiber matrix to impart UV photostability and to demonstrate mercury ion sensing.

**Figure 1:**
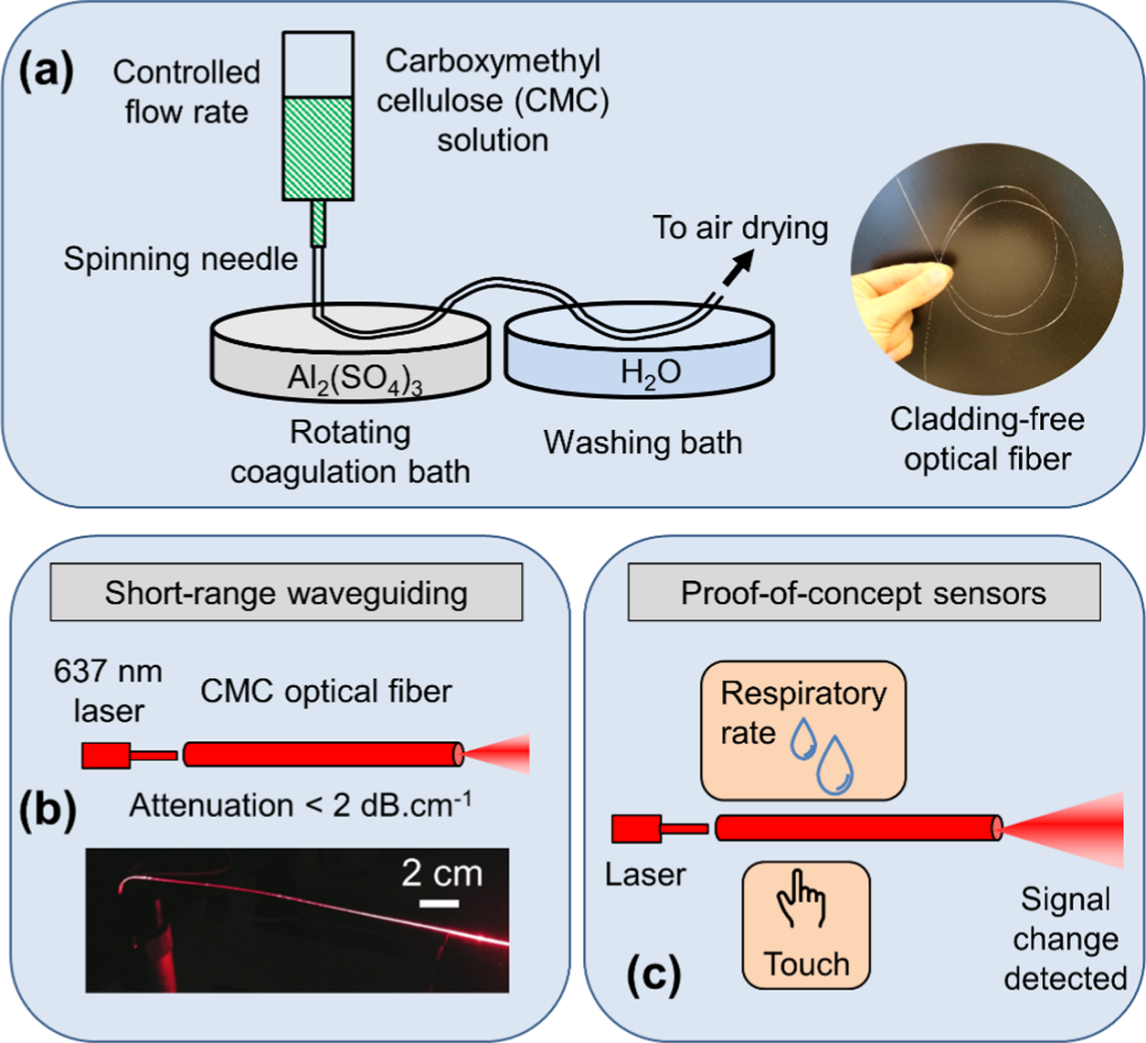
Schematic drawings describing the contents of the work. (a) CMC fiber manufacturing process, (b) short-range light transmission measurements, and (c) sensing experiments using fibers.

In this work, we present OFs fabricated using carboxymethyl cellulose (CMC) via a wet-spinning process (Figure 1a). CMC is a well-known ether derivate of cellulose with substituent carboxymethyl (−CH_2_COOH) groups with salient features such as low-cost, high-water solubility, nontoxicity, biocompatibility, and crosslinking ability.^22−25^ Exploiting this feature, CMC hydrogels were cross-linked with Al^3+^ ions via extrusion of hydrogel filaments directly into an aqueous aluminum sulfate solution. This facile fabrication process yields strong, transparent fibers while requiring short coagulation time.^26,27^ The properties of the prepared CMC OFs were investigated in terms of morphology, light attenuation (Figure 1b), and transmission spectra. Further, we demonstrate the application of cladding-free CMC OFs in touch sensing and respiratory rate monitoring (Figure 1c). Furthermore, we present a simple approach to impart water tolerance to CMC fibers via heat treatment and demonstrate high-speed signal transmission through OFs in both air and water media.

## Experimental Section

### Materials

A medium viscosity grade of sodium carboxymethyl cellulose (CMC) was procured from Merck KGaA, Germany, in powder form (Product name: C488). The degree of substitution for the used CMC was 0.8, and the molecular weight was approximately 250 kDa. A measured amount of CMC powder was slowly added to deionized water heated to 60 °C under constant stirring for 2 h to make a 5% w/w solution. The concentration of the CMC solution was selected such that the viscosity of the solution was suitable for the wet-spinning process.

Aluminum sulfate octadecahydrate (Al_2_(SO_4_)_3_·18H_2_O) was purchased from Merck KGaA, Germany, in powder form. The power was dissolved in deionized water to prepare a 0.5 M solution, which acted as a coagulating medium during fiber spinning.

### Methods

#### Fabrication of Optical Fibers from CMC Using Wet Spinning

Optical fibers from CMC were fabricated using the wet-spinning method, where a viscous solution of CMC was spun into a coagulation bath to produce fibers (apparatus shown in Figure S1 in the Supporting Information). Prior to fiber fabrication, all CMC solutions were deaerated using an asymmetric vacuum-aided centrifuge (SpeedMixer DAC 600, Synergy Devices Ltd., U.K.). First, a 5% w/w aqueous solution of CMC was carefully filled into a syringe (60 mL Luer-Lok, BD Plastipak) while avoiding any air entrapment into the solution. Next, a spinning needle (spinneret) of standard size was installed on the syringe tip (Hamilton Co.) to spin fibers of controlled diameter. Then, the filled syringe was mounted on a programmable syringe pump (NE-4000, New Era Pump Systems Inc.) with dispensing accuracy of ±1%. The outflow rate of the CMC solution from the syringe was selected according to the chosen spinning needle size. The CMC solution was extruded as fiber from the syringe in vertical configuration into a coagulation bath filled with aqueous 0.5 M Al_2_(SO_4_)_3_ solution. The distance between the needle tip and the coagulation bath was approximately 1 cm. Upon entering the bath, the CMC solution formed a hydrogel cross-linked with Al^3+^ ions, and continuous fibers of up to 100 cm length were produced in one spinning run. Further, the fibers were passed through a washing bath containing deionized water to wash away excess Al_2_(SO_4_)_3_ solution from the fiber surface. Lastly, the fibers were dried by hanging them vertically under tension (fiber ends were glued) to ensure dimensional stability upon drying. Post preparation, all fiber samples were conditioned at 23 °C and 50% relative humidity.

#### Characterization of Optical Fibers

Fiber thickness was measured using an L&W Micrometer 051 (Lorentzen & Wettre Ab, Sweden). The measured thickness values were confirmed using scanning electron microscope (SEM) images. SEM imaging of the fibers was performed using a field emission scanning electron microscope (FE-SEM) (Carl Zeiss Merlin, Germany) with a secondary electron detector at an acceleration voltage of 3.0 kV and a probe current of 60 pA. Sample surfaces were sputter-coated with a thin gold–palladium layer before imaging, and all images were taken at 2048 × 1536 pixel resolution. Further, energy-dispersive X-ray analysis (EDX) on the fiber samples was performed to determine their elemental composition. The spectra of sputtered metals were subtracted from the EDX data.

Attenuated total reflection Fourier transform infrared spectroscopy (ATR-FTIR) was performed on CMC films prepared via solvent casting to achieve a basis weight of 100 g/m^2^. The measurements were performed using a Thermo Scientific Nicolet iS50 FTIR spectrometer with an ATR diamond (Thermo Scientific). The ATR spectra were collected at room temperature in absorption mode in the wavelength range of 400–4000 cm^–1^ as the average of 64 scans with 4 cm^–1^ resolution (data shown in Figure S7a in the Supporting Information).

Mechanical properties of the fibers were measured using a Lloyd LS5 materials testing device (AMETEK Inc.) equipped with a load cell of 100 N. Tensile stress was applied to fiber samples of 30 mm length at a fixed rate of 3 mm/min. At least five measurements were performed for each sample, and the results are shown in Figure S3 in the Supporting Information.

#### Light Transmission and Attenuation Measurements

Light attenuation was measured with 637 nm laser light (Thorlabs, S4FC637) that was coupled to a single-mode (SM) fiber (9/125 μm core/cladding diameters). Transmitted signals were collected using a multimode (MM) optical fiber (105/125 μm core/cladding diameters) to a photodetector (Thorlabs, 120C) equipped with a power meter interface (Thorlabs, PM101). Micromanipulators (Melles Griot Inc.) were employed to ensure alignment between sample CMC fibers and glass fibers (Figure S4). A MM fiber with a smaller diameter than the sample CMC fiber was used to improve measurement accuracy. This avoided both direct light coupling from the light source to the detector and scattered light detection from cladding-free fibers. Measured signals were processed with LabView software.

#### Halogen Lamp and an Optical Spectrum Analyzer for Transmission Spectrum Measurements

Transmission spectra of CMC fiber samples were measured with the same micromanipulator setup as used in light transmission measurements (Figure S4 in the Supporting Information). The light source was changed to a halogen lamp (HK-2000-HP, Ocean Optics Inc.) and the detector to an optical spectrum analyzer (OSA) (AQ-6315A, Ando Electric Ltd.). A MM optical fiber (Thorlabs, 105/125 μm core/cladding diameters) was coupled from the halogen lamp to the CMC fiber, and transmitted light was collected with a MM optical fiber (Thorlabs, 400/425 μm core/cladding) and directed to the OSA. Light transmission spectra of the fiber samples were measured in the 350–1750 nm wavelength range with 10 nm resolution.

#### Respiratory Rate and Touch Sensor Measurements

**Figure 2:**
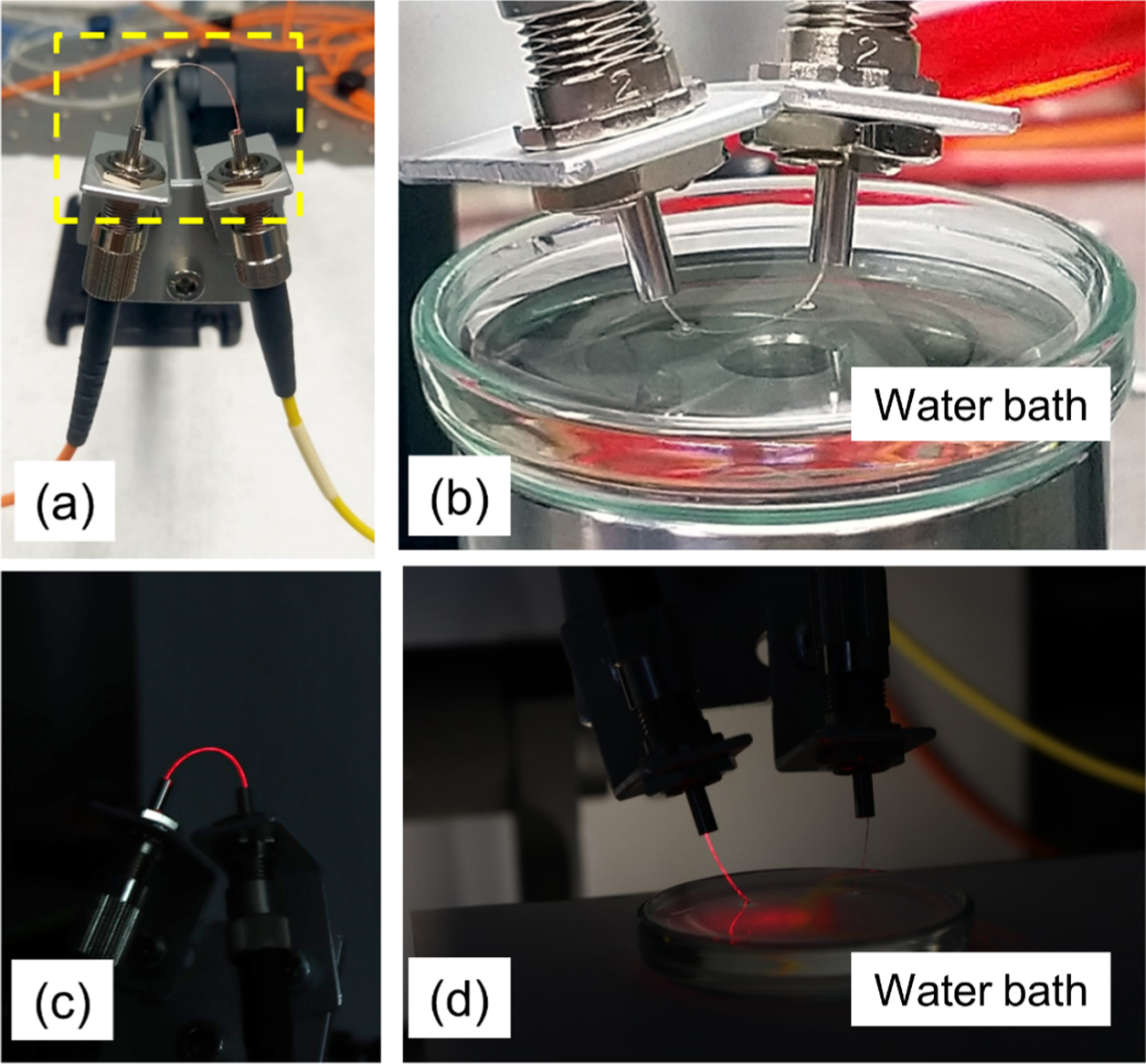
(a) Light coupling to the CMC fiber loop (yellow box placed to aid the reader). (b) Signal transmission under water with the heat-treated CMC fiber loop. Subfigures (c,d) show the same setups with fiber loops in the dark with red laser coupled to CMC fibers.

Micromanipulators used in the attenuation measurement setup were changed to FC/PC type optical fiber connectors (Figure 2a) in respiratory rate and touch sensor measurements. The principle of respiratory rate measurement was the change in light transmission upon moisture sorption–desorption by the CMC fibers. Upon exposure to breathing, moisture is adsorbed on the fiber surface, forming a temporary cladding that alters the refractive index on the fiber surface. This leads to a change in the light transmission through the fiber. Similarly, the desorption of the moisture from the fiber surface also changes the signal. Hence, if the sorption–desorption of moisture can occur rapidly in between breathing cycles, the respiratory rate can be detected using optical fiber. Touch sensing is enabled by cladding-free fibers because light escapes from the fiber core when an absorbing material or a material with a higher refractive index touches the fiber surface.

For this purpose, CMC sample fibers were coupled with FC/PC adapters and ferrules (Thorlabs, SF340-10, 340 μm hole diameter) to the glass fibers that also had FC/PC connectors. Transmitted light intensity from the samples was maximized using a MM optical fiber (Thorlabs, 400/425 μm core/cladding, FC/PC connectors) that had a larger diameter than the CMC fibers. The laser was changed to a 1050 nm superluminescent light-emitting diode (Thorlabs, S5FC1050P) because attenuation was slightly lower in the infrared (IR) range. The same photodetector was used in attenuation measurements. It was ensured that the optical coupling did not vary during the respiratory rate or the touch sensor measurements by fixing contacts between the measurement glass fibers and the tested CMC fibers.

#### Short-Range Broadband Signal Transmission Using CMC Fibers

**Figure 3:**
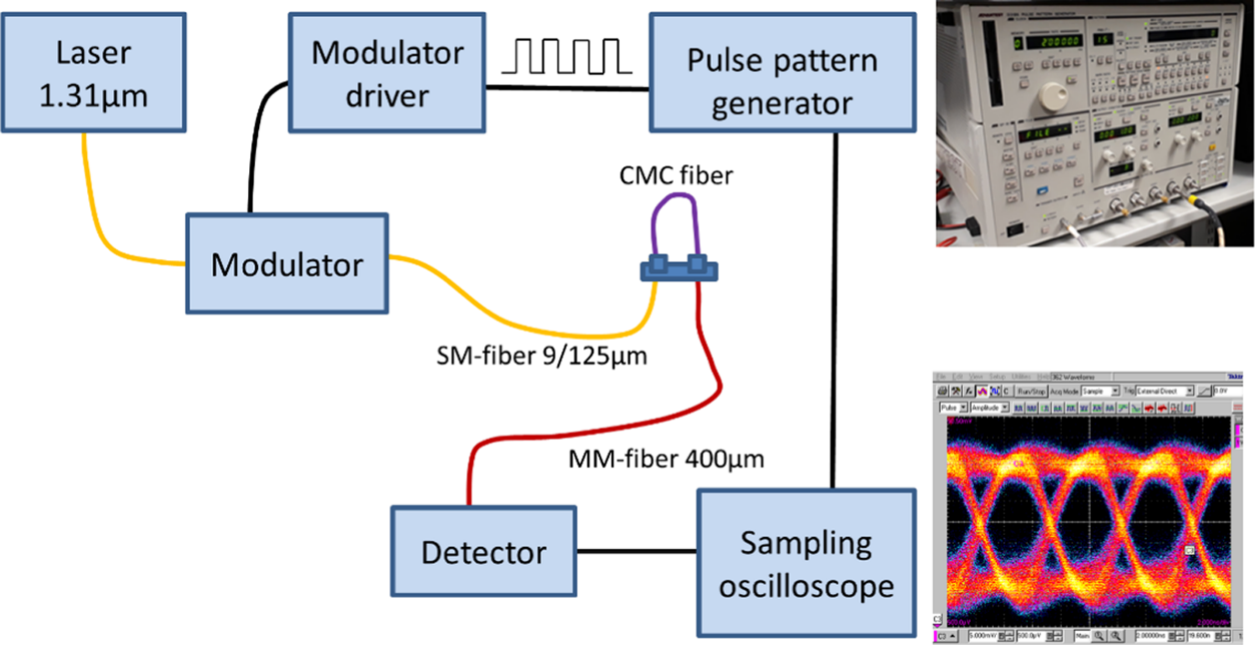
High-speed optical signal transmission measurement setup for CMC fibers.

To demonstrate short-range broadband signal transmission, 16G CMC fiber samples were used. The layout of the setup is shown in Figure 3. Light from a tunable laser (Santec TSL-510, Japan) at 1310 nm wavelength was modulated using an external modulator (OC-192 JDSU) and coupled to the CMC fiber with a 9/125 μm (core/cladding) SM optical fiber (Figure 3a). The light output from the CMC fiber sample was collected via a 400/425 μm MM glass fiber to a fixed gain amplified large-area photodetector (PDA05CF2 InGaAs Thorlabs). A 150 Mbit/s nonreturn to zero (NRZ) pseudorandom bit sequence (*N* = 2^15^ – 1) data pattern was created using a pulse pattern generator (Advantest D3186, Japan), and the output of the photodetector was connected to a sampling oscilloscope (CSA8000 Tektronix) that was triggered by the signal from the pulse pattern generator. The recorded eye pattern was used to evaluate the functionality of the short-range CMC fiber link. Data transmission capability of the heat-treated CMC was also tested under water (Figure 2b).

## Results and Discussion

### CMC Optical Fibers

Cladding-free optical fibers were fabricated from aqueous CMC solutions using wet spinning. Prior to spinning, the rheological behavior of CMC solutions at 3 and 5% w/w concentration was studied. Both CMC solutions were found to be highly viscous and exhibited shear-thinning nature in the shear flow measurements (data shown in Figure S2 in the Supporting Information). The 5% CMC solutions exhibited gel-like behavior under oscillatory strain at constant angular frequency. On the other hand, at 3% concentration, the solutions behaved like a viscous fluid. Thus, 5% concentration was chosen for fiber spinning as the higher viscosity and gelling nature were expected to result in better shape fidelity of CMC fibers upon drying.

Subsequently, CMC hydrogel filaments were cross-linked using Al^3+^ ions by extruding them directly into a coagulation bath containing the Al_2_(SO_4_)_3_ solution. Fibers of different thicknesses (samples named 15G, 16G, and 22G; Table 1) were prepared to investigate the effect of fiber diameter on the mechanical and optical properties. The correlation between the spinning needle diameter and the resultant dry fiber diameter was studied prior to sample preparation. The final sample diameters chosen for the present work were based on the ease of coupling light using commercial glass optical fibers to study their optical characteristics and the ease of physical handling.

**Table 1:** Description of Prepared CMC Optical Fiber Samples

**sample code**	**spinning needle**	**needle I.D. (mm)**	**dope extrusion rate (mL/min)**	**fiber diameter (μm)**
15G	15G	1.372	4	323 ± 16
16G	16G	1.194	4	280 ± 37
22G	22G	0.413	4	125 ± 11

I.D.: Internal diameter (nominal).

**Figure 4:**
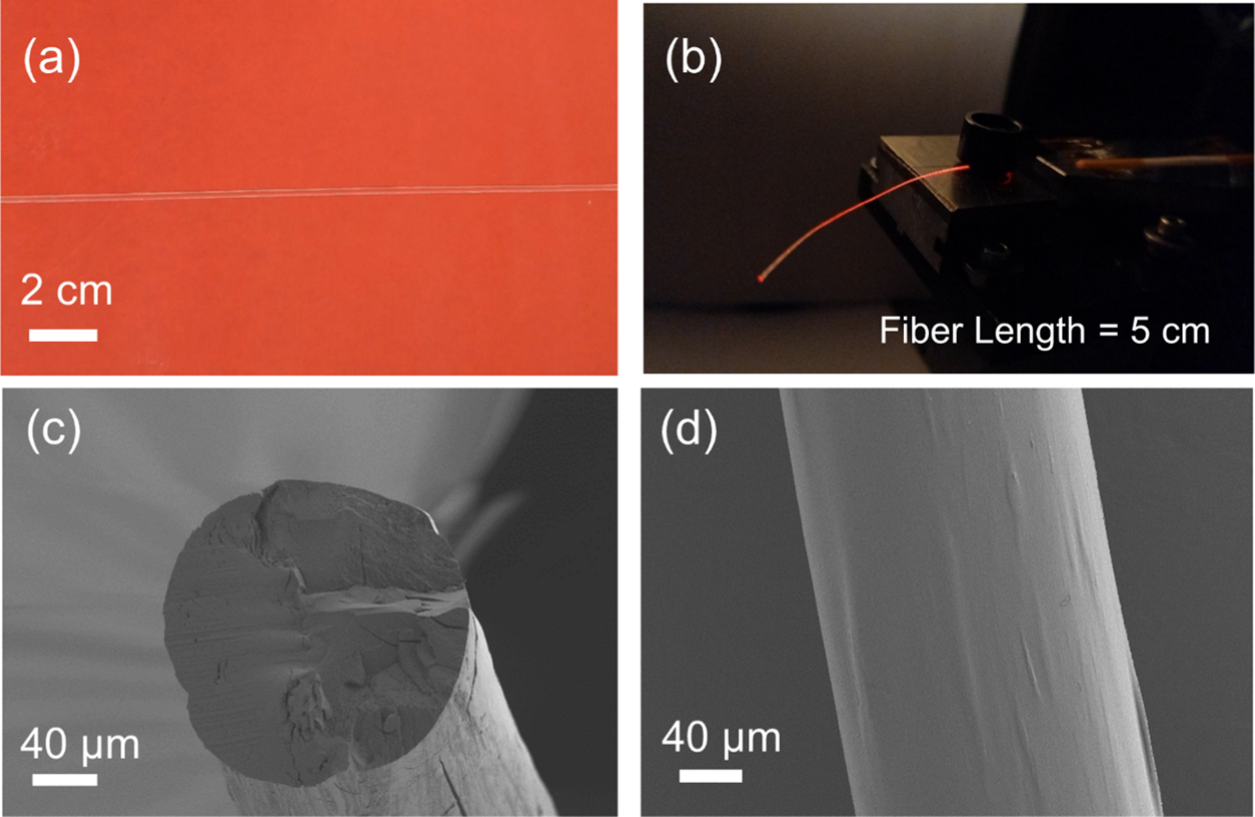
(a) Digital photographs of a CMC fiber (16G) placed on a red background and (b) the 16G sample when coupled to red laser light. Subfigures (c,d) show SEM micrographs of the 16G fiber sample cross-section and its surface, respectively (300X magnification).

The prepared CMC fibers were strong, flexible, and visually transparent. Even in the wet form, the fibers could be suspended vertically under tension, without breaking, to obtain dried solid fibers. The visual appearance of a selected CMC fiber sample (16G) is shown in Figure 4. It can be noted from Figure 4b that the CMC fiber is able to act as a waveguide and light is visible both on the fiber surface and at the tip. Light leakage from the fiber surface occurs due to intrinsic scattering and the absence of a cladding layer. In our fibers, the absence of a cladding layer is advantageous as the light transmission through the fiber is dependent on the refractive index of the surrounding media, thus offering the possibility to sense changes in the surrounding environment.^21^

Fiber morphology was investigated via SEM imaging. Figure 4c,d shows the images of the circular cross-section and the smooth surface of the 16G sample. Cross-sections for SEM imaging were prepared using a sharp razor blade, which caused cracks visible on the cross-section. Upon drying, slight twisting occurred in fibers as the diameter was increased above 200 μm. A similar observation has been reported elsewhere for methylcellulose fibers.^28^

EDX analysis was employed on fiber cross-sections to investigate the elemental distributions of aluminum ions in the fibers (Figure S5). A notable observation was that aluminum was distributed uniformly in the fiber bulk, even present near the center of the fiber. This confirmed that the fibers were thoroughly cross-linked and the coagulation time of 4–5 s was sufficient for Al^3+^ ions to diffuse to the core of the fibers.

The CMC fibers were also subjected to tensile tests to characterize their mechanical properties. The fibers were found to be strong but not stretchable as the tensile strength ranging from 125 to 150 MPa, and the elongation at break was 3–5% (data shown in Figure S3). The tensile strength values are comparable to those reported by Hynninen et al. for pure MC fibers (100–150 MPa), but the stretchability of CMC fibers is much lower (∼30–50% for MC fibers).^21^ This difference can be attributed to both the difference in the chemical structure of MC and CMC and the preparation method for the fibers. Hynninen et al. fabricated MC fibers by spinning pure solutions into an ethanol coagulation bath with 25 min coagulation time, followed by ambient drying. On the contrary, CMC fibers in our work were cross-linked, and the residence time in the coagulation bath was only 4–5 s followed by ambient drying. Here, MC contains substituent methyl groups that do not participate in intermolecular hydrogen bonding, but on the contrary, carboxymethyl groups in CMC can form hydrogen bonds, leading to tougher, nonstretchable fibers. However, the higher stiffness in the CMC fibers could be more influenced by the crosslinking involved in the preparation process. Ionic crosslinking is known to increase the stiffness of hydrogels, for instance, higher mechanical stiffness of CMC hydrogels has been shown upon crosslinking with various metal ions.^29^

### Optical Properties and Sensing Response

The preparation method of CMC fibers via Al^3+^ ion crosslinking is known in the literature.^27^ However, the potential of those as optical fibers has not been tapped yet. In the present work, emphasis was placed on investigating the optical properties of the prepared CMC fibers and demonstrating their utilization in sensing systems, namely, respiratory rate measurement and touch sensing. Short-range, high-speed signal transmission was also demonstrated in both air and water media.

### Light Transmission Spectrum and Attenuation Constant

In relation to the performance of optical fibers, one of the key aspects is their ability to transport light without intensity loss. GOFs are well-known for their lossless waveguiding feature where the attenuation constant (α) can be as low as 2 × 10^–6^ dB·cm^–1^.^30^ However, such low-attenuation constant is required for applications such as long-distance signal transmission, where the transmission length spans hundreds of kilometers. For sensing applications, the required transmission length could vary from only a few centimeters up to several meters. For instance, light delivery to organ scale lengths (∼10 cm) is required for biomedical applications such as imaging and phototherapy.^31,32^ Thus, a performance similar to GOFs is not necessary to achieve with cellulosic OFs.

**Figure 5:**
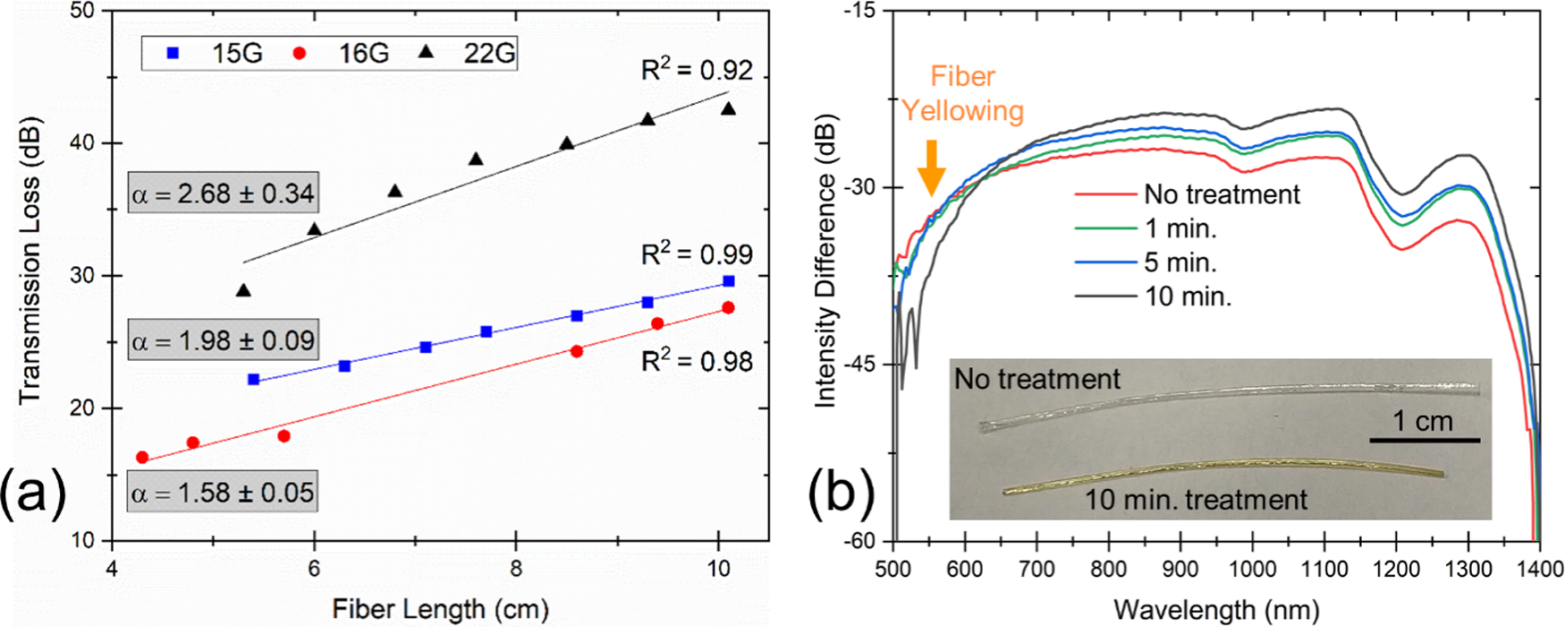
(a) Transmission loss measured as a function of fiber length using a visible 637 nm laser for three different CMC fiber samples. The data was fitted linearly, and the slope of the fitted straight line denotes the attenuation constant (α) for the fibers, measured in dB·cm^–1^. (b) Transmission spectra for 16G fiber samples with 0, 1, 5, and 10 min of heat treatment at 160 °C. The inset image shows the apparent yellowing of fiber after 10 min heat treatment.

In the current work, we measured the attenuation constant for CMC fibers using a 637 nm visible laser (10 mW intensity). Figure 5a illustrates the measurement results, where the best performing sample 16G exhibited α = 1.58 ± 0.05 dB·cm^–1^, while other CMC fiber samples exhibited slightly higher values, still below 3 dB·cm^–1^. If we compare the optical performance of the prepared CMC fibers to other cellulosic optical fibers, Orelma et al. had previously reported α = 6.3 dB·cm^–1^ at 1300 nm for fibers prepared from regenerated cellulose via dry-jet wet spinning.^19^ In a separate work, an attenuation value of roughly 1 dB·cm^–1^ (600 nm) was reported by Reimer et al. for bare regenerated cellulose fibers.^4^ Thus, the 16G sample in the current study shows roughly 4 times lower attenuation than that reported by Orelma et al. and 1.5 times higher than that reported by Reimer et al. However, it can be noted from the transmission spectra of the CMC fibers that the transmission level peaks in the IR region (Figure 5b). Hence, the CMC fibers can be expected to exhibit even lower α when measured in the IR range and be on par with state-of-the-art cellulose optical fibers. Furthermore, we compared the performance of CMC fibers to another class of flexible optical fibers, i.e., POFs. A commercially available PMMA fiber (X-ON, FDPF 4001 EH) was measured using the same setup, and α was found to be 0.0056 dB·cm^–1^, which is 3 orders of magnitude lower than current cellulose fibers.

### Heat Treatment for the Water-Resistant CMC Fiber

Cellulose materials are known for their highly hygroscopic and water-swelling nature.^33^ CMC, being an anionically charged cellulose derivative, exhibits an even higher water-swelling ratio than native cellulose as it facilitates osmosis.^34,35^ This water-swelling behavior can be utilized for sensing water^19^ or moisture,^36^ but it could also pose challenges for some sensing applications due to the presence of water in the fiber. Additionally, CMC fibers have poor wet strength and disintegrate when exposed to water for long periods. To avoid this problem, the CMC fibers were heat-treated at 160 °C for up to 10 min to impart water resistance to them. Fibers were found to become water resistant upon the heat treatment and turned yellow (Figure 5b). Yellowing of cellulose at elevated temperatures has earlier been attributed to the oxidative reactions that lead to the formation of chromophores.^37,38^ In the case of fibers, yellow coloration has been described previously for regenerated cellulose fibers elsewhere.^4^ Nonetheless, it was confirmed via ATR-FTIR spectroscopy and thermogravimetric analysis (TGA) that CMC does not undergo major chemical changes upon heat treatment at 160 °C for 10 min and also does not suffer extensive mass loss (Figure S7a,b in the Supporting Information). It must be noted that the heat treatment tests and further sensor demonstrations in this work have been performed using the 16G sample as it showed the best optical performance and easiest handling.

Light transmission through fibers was tested before and after the heat treatment at different treatment times of 0, 1, 5, and 10 min (Figure 5b). Fiber sample length was kept constant at 2.9 cm during transmission measurements to ensure proper comparison between the collected spectra. The total transmission range for the CMC sample was observed to be 550–1350 nm, and it is similar to the range reported previously for regenerated cellulose fibers.^19^ As the heat treatment time was increased, the transmission increased in the IR range (650–1350 nm) and the highest transmission increase was observed in the 1300 nm range. High attenuation was seen in the visible range under 500 nm both before and after heat treatment, but at a higher visible range 500–650 nm, the transmission decreased for heat-treated samples as the fibers became yellower in color.

### Respiratory Rate Sensing

**Figure 6:**
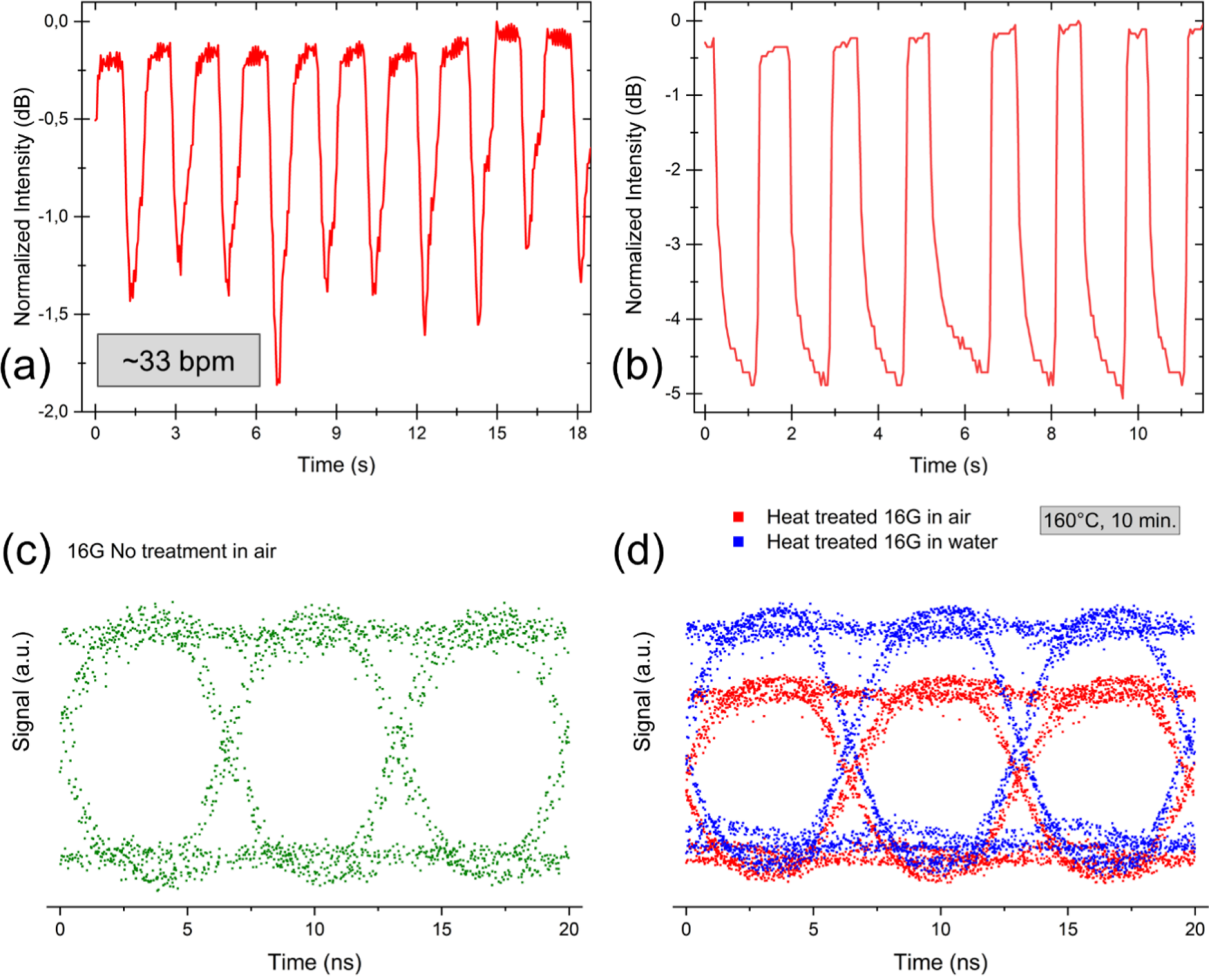
(a) Respiratory rate monitoring using the 16G fiber sample, where a respiratory rate of 33 breaths per minute (bpm) was measured and (b) touch sensing using 16G fibers. Panel (c) shows the eye diagram of optical signal transmission through 16G fiber in air and (d) eye diagram of heat-treated 16G fibers (160 °C, 10 min) in air and under water.

CMC fibers (16G sample) without any heat treatment were tested in a respiratory rate sensor application. Samples were exposed to breathing in a fiber loop configuration, as shown in Figure 2a. The measurement results are illustrated in Figure 6a, where the ground level of the signal was normalized to 0 dB to aid interpretation. The signal was found to decrease periodically upon exposure of the sample to breathing. This behavior can be explained by the water-interaction properties of CMC, where moisture from breathing was adsorbed rapidly by the fiber, causing an increase in the attenuation and decreasing the signal intensity. The moisture desorbed quickly from the CMC fiber surface, and the ground-signal level was regained in about 1 s. As shown in Figure 6a, the measured respiratory rate was about 33 breaths per minute (bpm). It is remarkable that in this demonstration, the optical fiber material itself is sensitive to moisture and acts as an active sensing component. Thus, any extra material is not needed to enhance the fiber sensitivity as described in literature previously, for instance, by Mathew et al., where agarose gel was filled inside a photonic crystal GOF.^15^

### Touch Sensor

CMC optical fibers were also tested as a touch sensor. Cladding-free fibers are sensitive to touching because light escapes from the fiber core when an absorbing material or a material with a higher refractive index touches the fiber surface. In such cases, signal change is a function of contact surface area. The measurements were performed using a 11.7 cm long CMC fiber (16G) with 1 cm fiber loop in the middle of the fiber, employing the same optical coupling as in the respiratory rate monitoring test (Figure 2a). The fiber loop was touched with finger 7 times in a span of 11 s. Figure 6b illustrates the results from the measurement where the ground level of the signal was normalized to 0 dB. The signal was found to decrease by ∼4.5 dB with every touch, and each touch was observed in the recorded signal. Interestingly, the signal level kept decreasing as long as the finger was placed on the fiber, probably because moisture was transferred from skin to fiber. After the removal of touch, the signal instantly recovered to the ground level. It must be noted that the sensitivity of CMC fibers is limited to touch and not pressure as the sensing principle is based on light leakage from the cladding-free fiber. Pressure sensing requires changes in the cross-section geometry as described previously elsewhere with thermoplastic silicone fibers.^39^

### Broadband Optical Signal Transmission

The maximum optical signal transmission capability of a 4 cm long CMC fiber (sample 16G) was tested in both air and under water. Due to the large diameter of 16G fiber samples, a wide-area detector must be used, whereby the bandwidth of the detector (150 Mbit/s) limits the achievable transmission speed. However, such a high signal transmission rate is already sufficient for many sensing applications; for instance, the used 150 Mbit/s rate is sufficient to run six Ultra HD 25 Mbit/s television channels in parallel. The 16G fiber samples were tested both before and after heat treatment. The measurements could not be performed in water for untreated fibers as they disintegrated rapidly upon wetting. However, upon heat treatment for 10 min at 160 °C, the fibers could be tested under water. The rationale behind testing signal transmission under water is that biocompatible CMC fibers could possibly be used for biological sensing applications where liquids are often present. The fibers could then also be used to measure liquid properties such as refractive index and concentration.^40^Figure 6c,d illustrates the measured eye patterns from the signal transmission measurements in arbitrary units (a.u.) as the measured signal levels before and after heat treatment are not directly comparable due to change in optical coupling. Nevertheless, the signal level should increase after heat treatment since the wavelength used in these measurements is 1310 nm. Moreover, the transmission spectra show that the total transmission of the CMC fibers increases post heat treatment in the 1310 nm range (Figure 5b).

Post heat treatment, the optical coupling remained constant, and hence, signal levels can be compared between air and water (Figure 6d). The length of fiber immersed in water was nearly 1 cm. The signal level increased slightly under water due to water acting as a cladding layer on the CMC fiber, which smoothened the optical roughness of the fiber surface and decreased the light scattering. An exciting feature of these results is that in all tested cases, constant binary levels 1 and 0 are well separated and the transition between the levels is clearly seen from the measured eye patterns, which corresponds to minimal signal distortion in the data link. Thus, the demonstrated 150 Mbit/s transmission rate shows good optical performance and is sufficient for sensing applications requiring high transmission rates. For instance, in an optogenetics application, mice were subjected to 3 ms long optostimulation pulses at 67 Hz frequency (473 nm blue laser diode) to treat Parkinson’s disease.^16^

## Conclusions

In this work, we fabricated cladding-free optical fibers using carboxymethyl cellulose (CMC) and demonstrated their utilization in sensing applications. Fibers were prepared via the wet spinning of aqueous CMC hydrogels into a coagulation bath containing an aqueous solution of aluminum sulfate. We demonstrated the tunability of the fabrication process by producing fibers of different diameters ranging from ∼125 up to ∼320 μm. Although this CMC fiber spinning process is documented in the literature, the utilization of CMC fibers as optical fibers has not been studied. Therefore, our work demonstrates the utilization of CMC fibers in novel photonic applications in environmental sensing and short-range data transfer.

The optical properties of CMC fibers were characterized with attenuation and transmission spectrum measurements. The transmission window of CMC fibers was found to be 550–1350 nm, and attenuation constant values ranged from 1.6 to 2.7 dB·cm^–1^ when measured at 637 nm. The prepared CMC fibers were used in a cladding-free configuration to be able to act as an active sensing component in respiratory rate monitoring and touch sensing. The respiratory rate sensor utilized the fast moisture sorption kinetics of CMC, and a respiratory rate of 33 breaths per minute was measured with good accuracy. Correspondingly, the touch sensor also took advantage of the cladding-free fiber structure, where light leakage during touching caused an attenuation increase of ∼4.5 dB and enabled sensing.

Lastly, the optical signal transmission performance of CMC fibers was tested using a 150 Mbit/s signal (1310 nm) over a short distance (4 cm). The fibers successfully transmitted the high-speed signal. Further, heat-treated CMC fibers (160 °C, 10 min) were tested for signal transmission, and high-speed transmission was possible both in air and water media.

The biodegradable and biocompatible nature of CMC makes it a suitable candidate for sensing applications in fields such as *in vivo* biological sensing, tissue health monitoring, cell therapy, and optogenetics. The utilization of CMC fibers in the three sensing applications presented in the current work demonstrates the possibility of their use as active sensing components in both air and in liquid media and opens the possibility of further exploration. Future work on the topic could focus on optimization of the fiber spinning process parameters to achieve lower light attenuation values. Different biopolymers can be explored for preparing optical fibers, and the effect of cladding materials can be studied. Lastly, aluminum ion crosslinking used in the study might affect the biocompatibility of CMC fibers; hence, the use of alternative crosslinking agents such as calcium ions could be investigated.

## Supporting Information Available

Supporting Information showing the used experimental setups and material characterization (PDF)
