# Supplementary material for: Carboxymethyl Cellulose (CMC) Optical Fibers for Environment Sensing and Short-Range Optical Signal Transmission
Source: ACS Appl Mater Interfaces. 2022 Jan 8;14(2):3315–23. doi: 10.1021/acsami.1c22227 (PMC8778620; doi:10.1021/acsami.1c22227)
Supplement: Supplementary file 1 — am1c22227_si_001.pdf [file am1c22227_si_001.pdf]

Supporting Information

Carboxymethyl Cellulose (CMC) Optical Fibers for Environment Sensing and Short-range Optical Signal Transmission

Aayush Kumar Jaiswal[1], Ari Hokkanen[2], Markku Kapulainen[2], Alexey Khakalo[1], Nonappa[3], Olli Ikkala[4], Hannes Orelma[1]*

[1]Biomaterial Processing and Products, VTT Technical Research Centre of Finland Ltd., Tietotie 4E, 02044 Espoo, Finland

[2]Microelectronics, VTT Technical Research Centre of Finland Ltd., Tietotie 3, 02044 Espoo, Finland

[3]Faculty of Engineering and Natural Sciences, Tampere University, P.O. Box 541, 33101 Tampere, Finland

[4]Department of Applied Physics, Aalto University, P.O. Box 15100, 00076 Espoo, Finland

Email: aayush.jaiswal@vtt.fi

KEYWORDS: cellulose, optical fibers, sensors, respiratory sensors, green photonics, biosensors

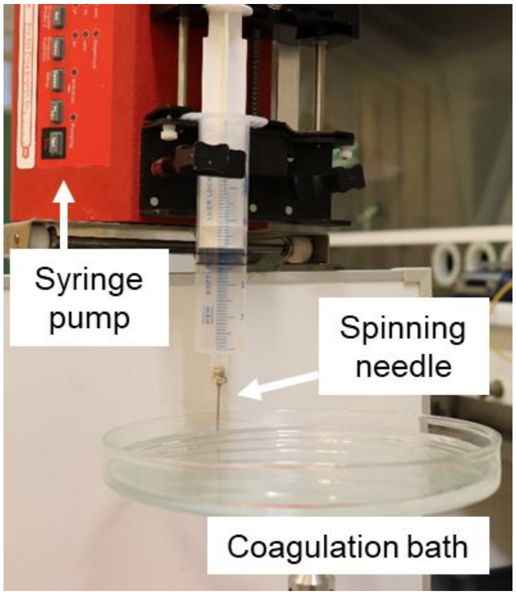

Figure S1. Image of the CMC fiber preparation setup. Fibers were fabricated via wet-spinning in a vertical configuration.

Figure S1 shows an image of the fiber spinning step. The syringe pump pushes the spinning dope contained in the syringe through a spinning needle and as a filament into a coagulation bath. The coagulation bath rotates at slow speed to enable fiber collection for further washing and drying.

Prior to fiber spinning, rheological behavior of CMC aqueous solutions was analyzed and the properties of 3% and 5% CMC solutions were compared. Rheology measurements were performed using a stress-controlled rotational rheometer Physica MCR 301 (Anton Paar GmbH) using a vane-cylinder geometry (STV-22, Anton Paar GmbH; vane span: 22 mm, cup diameter 29.29 mm). The

vane was fully immersed into the cylinder filled with the sample fluid while maintaining 1 mm vertical gap between the vane and the cylinder. Firstly, forward shear sweeps were performed in the shear rate range of 1-300 s^{-1} (Figure S2a). Higher shear rates could not be achieved using the measurement setup due to the onset of Weissenberg effect (rod climbing). Subsequently, to ascertain the gelling nature of the CMC solutions, oscillatory amplitude sweeps were performed in the strain range of 1-200% while keeping the angular frequency constant at 10 rad.s^{-1}. Storage moduli (G') and loss moduli (G'') of 3% and 5% solutions were measured and are shown in Figure S2b.

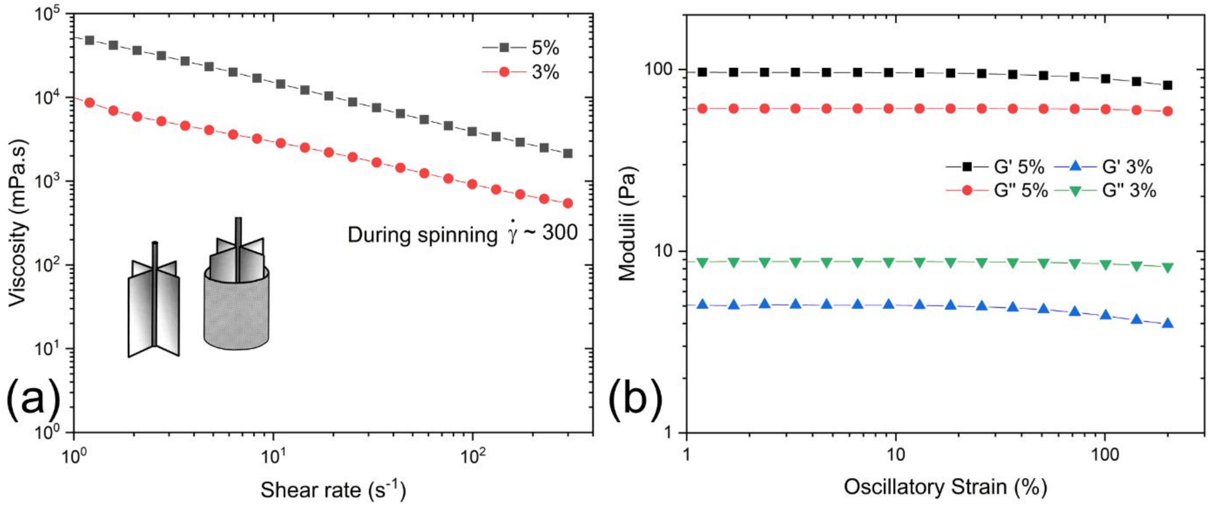

Figure S2: Rheological behavior of CMC aqueous solutions at 5% and 3% w/w concentration. (a) shows forward shear flow curves and (b) shows amplitude sweep curves at fixed angular frequency

of 10 rad/s. Both measurements were performed using a cylinder-vane geometry (depicted in inset of subfigure (a)).

Both 3% and 5% CMC solutions exhibited high viscosity along with a shear-thinning nature (Figure S2a). The shear rate at the wall applied on the solutions during extrusion from the spinning needle was approximated using the Hagen-Poiseuille equation:

$$\dot{\gamma} = \frac{32\dot{Q}}{\pi D^3} \qquad (1)$$

where $\dot{\gamma}$ is the shear rate (s^{-1}), Q is the volumetric flow rate ($m^3.s^{-1}$), and D is the inner diameter of the spinning needle (m).

It must be noted that the Hagen-Poiseuille equation holds valid only for Newtonian fluids in an ideal flow whereas the CMC solutions show a non-Newtonian behavior. However, the equation was used to calculate an approximate value of shear rate induced on the fluid during the spinning process. For 15G and 16G spinning needles, the shear rate at the wall can be calculated to be 268 and 399 s^{-1} using Equation (1). At such shear rates, the apparent viscosity of the CMC solutions are already 10 times lower than the zero-shear viscosity.

From Figure S2b, it can be observed that the 5% CMC solution exhibited highly gel-like nature (phase angle = 32°) and there was no crossover point observed even until the exertion of 200%

strain on the sample. However, the gelling nature was absent in the 3% solution where the loss modulus was found to be higher than the storage modulus (phase angle = 61˚), thus indicating that the solution concentration lies below the gel-point concentration.

Figure S3 shows the stress-strain curves for the 15G, 16G, and 22G fiber samples measured in the tensile direction. The thinner 16G fibers (280 ± 37 μm diameter) elongated less than the thicker 15G fibers (323 ± 16) before failure and were slightly weaker in terms of ultimate tensile strength. The thinnest 22G fiber (~125μm diameter) were extremely weak in comparison to the other samples and hence not used during sensing experiments.

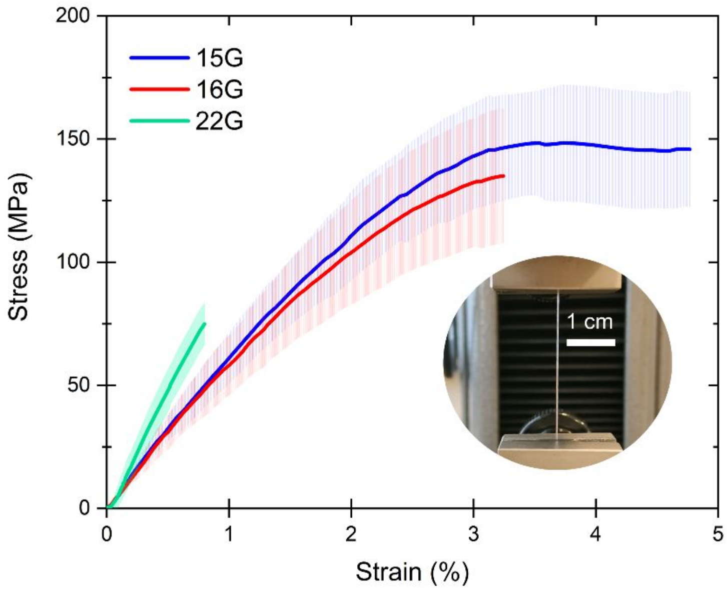

Figure S3. Stress-strain curves for 15G and 16G fiber samples along with their respective standard deviations.

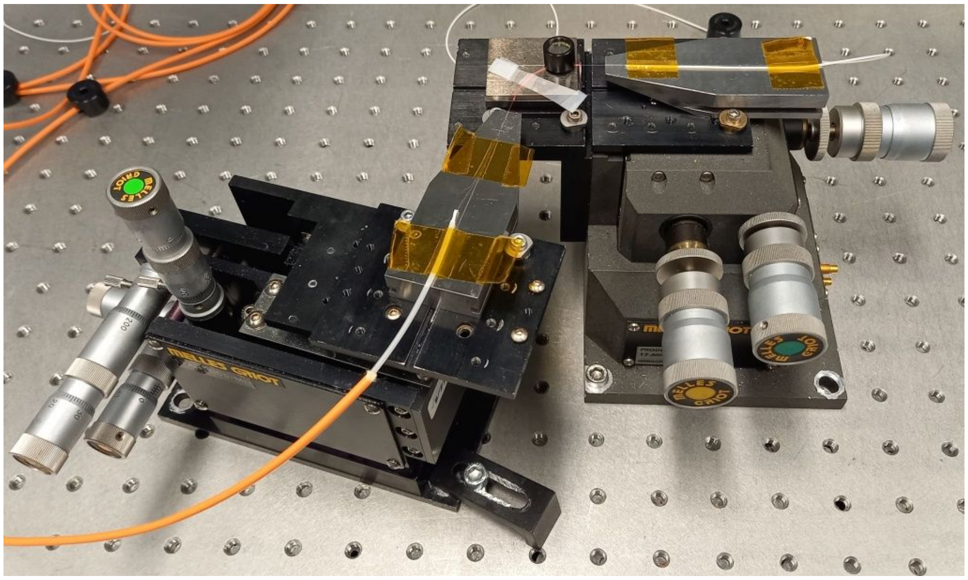

Figure S4. Experimental setup for light transmission and attenuation measurements.

Figure S4 shows the experimental setup for light transmission and attenuation measurements. The measurement protocol has been described in the Methods section of the main text.

Figure S5 and S6 show the results obtained from the EDX analysis of the fiber samples. The elemental analysis naturally showed that carbon and oxygen are the most dominant elements, along with the presence of sodium and aluminum. Sodium presence is explained by the fact that a sodium salt of CMC was used in the study. Aluminum was found to be homogenously distributed in the fiber bulk, even near the center, indicating that Al^{3+} ions were able to penetrate rapidly into the CMC hydrogel bulk and participate in cross-linking the hydrogel.

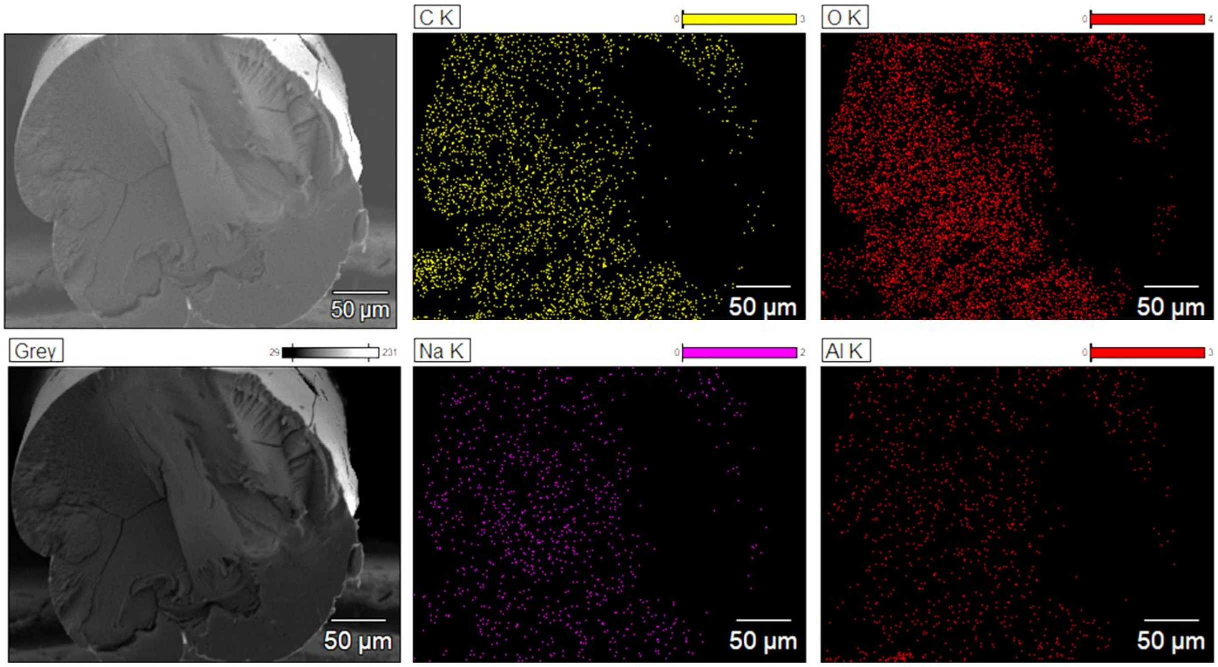

Figure S5. SEM-EDS elemental maps for the 16G fiber sample. The identified elements which are mapped are carbon, oxygen, sodium, and aluminium. The SEM image is also shown in greyscale.

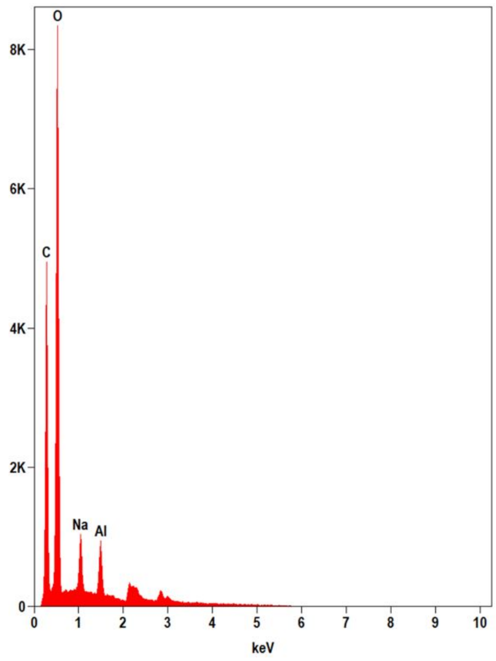

Figure S6. Relative elemental concentrations measured from the cross-section of the 16G fiber sample via EDX.

Characterization of the CMC used in the study was also performed, using ATR-FTIR spectroscopy and Thermogravimetric Analysis (TGA) and Dynamic Scanning Calorimetry (DSC). The experimental protocol for ATR-FTIR has been described in the main text under the Methods section. TGA and DSC measurements were performed in open alumina pans surrounded by air in the range of 50-600 ˚C and 50-300˚C, respectively. The heating rate was fixed at 5 ˚C/min and the instrument was a Netzsch STA 449 F1 Jupiter (Netzsch-Gerätebau GmbH, Germany) thermogravimetric analyzer. The measurements were performed at least twice.

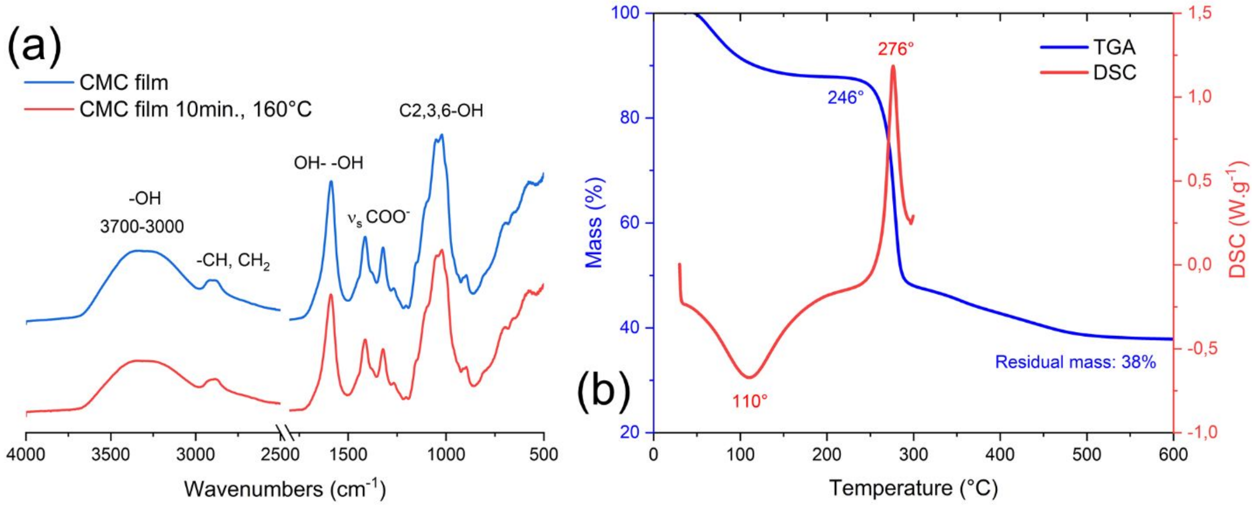

Figure S7. (a) FTIR spectra for CMC films (100 g/m^2) before and after heat treatment at 160˚C for 10 minutes. (b) TGA and DSC curves for CMC powder measured in air.

Figure S7a shows the FTIR spectra for 100 g/m^2 films prepared via solvent casting of CMC solutions at a basis weight of 100 g/m^2. Spectra were taken before and after heat treatment (160˚C for 10 min.) of the film to observe any chemical changes in CMC. All characteristic bands of CMC are present in the spectra both before and after treatment. The bands at 1600, 1416, 1324 cm^{-1} correspond to the COO^- vibrations. No spectral change was observed upon the heat treatment.

Figure S7b shows the TGA and DSC curves for CMC powder in air. The TGA curve showed two decaying periods, first upon the loss of moisture from the sample, and the second upon combustion of volatiles in the sample. The endothermic peak of the DSC curve was seen at ~110

˚C which marked complete dehydration of the sample. Upon further heating, the curve starts moving towards the exothermic direction where the exothermic peak was seen at ~276 ˚C.

Figure S8 shows SEM images of fiber cross-sections for 15G and 22G samples where twisting in the fiber is apparent in the image of the 15G fiber. It must be noted that twisting occurs intermittently, arising from shrinkage forces experienced by the hydrogel while drying if the fiber formation is non-uniform. As the fiber diameter decreases i.e., in the case of the 22G fibers (smallest diameter in the current study), the twisting tendency seems to reduce.

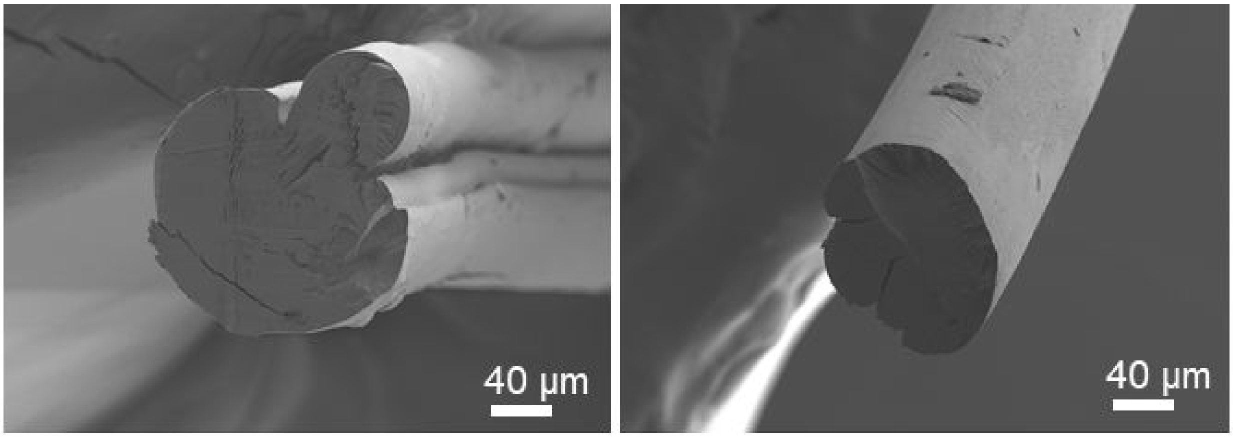

Figure S8. SEM images of fiber cross-sections for 15G (left) and 22G (right) samples at 270X magnification.
